# Detecting and Mitigating Wind Turbine Clutter for Airspace Radar Systems

**DOI:** 10.1155/2013/385182

**Published:** 2013-12-05

**Authors:** Wen-Qin Wang

**Affiliations:** School of Communication & Information Engineering, University of Electronic Science and Technology of China, Chengdu 611731, China

## Abstract

It is well recognized that a wind turbine has a large radar cross-section (RCS) and, due to the movement of the blades, the wind turbine will generate a Doppler frequency shift. This scattering behavior may cause severe interferences on existing radar systems including static ground-based radars and spaceborne or airborne radars. To resolve this problem, efficient techniques or algorithms should be developed to mitigate the effects of wind farms on radars. Herein, one transponder-based mitigation technique is presented. The transponder is not a new concept, which has been proposed for calibrating high-resolution imaging radars. It modulates the radar signal in a manner that the retransmitted signals can be separated from the scene echoes. As wind farms often occupy only a small area, mitigation processing in the whole radar operation will be redundant and cost inefficient. Hence, this paper uses a transponder to determine whether the radar is impacted by the wind farms. If so, the effects of wind farms are then mitigated with subsequent Kalman filtering or plot target extraction algorithms. Taking airborne synthetic aperture radar (SAR) and pulse Doppler radar as the examples, this paper provides the corresponding system configuration and processing algorithms. The effectiveness of the mitigation technique is validated by numerical simulation results.

## 1. Introduction

Due to the threat of climate change, countries around the world are working for the production of renewable energy. One such renewable energy source is the power available from the wind [1–4]. However, erecting a wind farm involves many considerations including consultation with various civil or military aviation interests. They may raise objections to a proposed wind farm for various reasons. One common objection is that wind turbine may be a threat to the safety of low-flying military aircrafts. Another objection is that wind turbine may appear as a strong radar echo [5–8]. This echo may distract the radar from the target echoes which are its main interest and can reduce the effectiveness of the radar by masking genuine target echoes [9–11]. Hence, wind farm may be a threat to existing radar systems [12–18]. Consequently, there is a conflict of interest between the desire to encourage wind farm development as a renewable energy source and the desire to maintain the performance of existing radar systems.

As the velocity of the blade tips can reach up to 75 m/s, the rotating wind turbine blade will impart a Doppler frequency shift to any radar signal reflecting off the blade. In this case, the radar's moving target indication (MTI) depending on the designed thresholds in the processor may detect it as a nonstatic target. In wind farms, variation in the wind direction at the turbine, the precise position of the blade in its rotation as the radar beam illuminates it, the pitch of the blade, and other factors may cause the amplitude and size of the radar echoes to fluctuate from one antenna rotation to another. At sites with a single turbine, any radar echo from the rotating turbine blades may stay in the same location on the radar screen. However, at sites with more than one turbine, the radar may illuminate two and more turbines during one antenna sweeping time. This will result in the images on the radar screen moving about within the area of the wind farm over time. The extent to which this will happen depends on, amongst other factors, the minimum distance between objects that the radar can detect. Moreover, wind farms will have a much more severe effect on the radar systems when the wind farms are located within uncontrolled airspace than when they are situated within controlled airspace. In uncontrolled airspace, aircrafts may fly without contacting any air traffic control agency. In this case, aircrafts can only use a radar to provide the navigation information.

Additionally, wind farms can cause degradation to other existing electronic systems besides radar systems, especially for television terrestrial broadcasting services [19]. Some studies have investigated this degradation in several countries, mainly in USA, United Kingdom, Denmark, Spain, and The Netherlands. The main effect of the interference from wind turbines on television services is a static ghost in the picture or a cyclic variation of the brightness in the picture [20, 21]. This is unsatisfactory to wind energy industry because it is hard for the aviation safety authorities to give a clear and well-founded decision on whether a particular proposed wind farm presents a safety issue or not; hence, it restricts the areas available for wind farm development.

Although specified wind farm configurations can be employed for the wind energy systems [22], it is necessary to mitigate the effects of wind farms on radar systems to maintain and improve the radar surveillance [13]. Several groups have suggested some mitigation measures [23–27]. A typical proposal is to modify the inside of the blades with layers of circuits and reflectors that can reduce the strength of the radar return from the blades. However, this technique is a highly frequency-specific method. It is necessary to distinguish and mitigate the impacts of wind farms on radar systems that were not anticipated in the original design specifications for either radar or wind farm engineers.

To mitigate the effects of wind farms on radar systems, this paper presents a transponder-based technique. The novel transponder was proposed by Weiß [28] for calibrating high-resolution imaging radars. This transponder using a wideband voltage-controlled attenuator (VCA) modulates the radar signal in a manner that the retransmitted signals can be separated from the scene echoes. This information can then be used to determine whether the radar is impacted by the wind farms. Thereafter, the effects of wind farms can be mitigated by subsequent signal processing algorithms.

The remaining sections are organized as follows. In Section 2, related work on the mitigation techniques of wind farms is introduced and motivation of this paper is outlined. In Section 3, the transponder-based wind turbine clutter detection is described. Next, the mitigation processing algorithms are presented in Section 4. Finally, Section 5 concludes the whole paper.

## 2. Background and Motivation

When a radar is operating, all the illuminated objects reflect some of the energy back to the radar receiver. The reflected signals are modified in various ways depending on the reflection process. Radar just exploits these modifications to differentiate between certain types of objects. By designing specific processing algorithms, the radar can differentiate between stationary targets and moving targets. This enhances the detection of desired targets and suppresses the detection of the terrain surface, buildings, and so forth. If the motions of the illuminated turbine blades lie in similar velocity bands to the desired moving targets, they cannot be distinguished from the moving targets. In this case, Doppler filtering is not an effective method. It should be accepted that these signals will pass into the radar receiver and other methods should be applied in subsequent signal processing steps.

Although the effects caused by the wind turbines are difficult to quantify, it is well recognised that wind farms can degrade the performance of radar systems significantly. In summary, there are two significant features. The first is the relative strong turbine echoes and the second is the Doppler frequency shifts caused by the movement of the wind turbine. Consequently, several potential effects are [29–33] as follows: (1) suppression of the radar sensitivity: high signal levels may bury some small signals reflected by the desired targets; (2) reduction in the ability to track target close to the wind farms: large signal levels reduce the capability of the radar to resolve closely spaced objects, and the desired targets may be “lost” in the vicinity of the wind farm; (3) increased false detection: if the radar cross-section (RCS) of a wind turbine is large [34], “ghost” may be generated in the subsequent radar signal processing [35]. Certainly, the effects of wind farms on radars depend on the internal radar design and the particular wind turbine characteristics, but they are often not anticipated in the original design specifications. Therefore, efficient mitigation methods are required.

Most published mitigation techniques are usually based on improving the radar's ability to discriminate between wanted and unwanted targets [36, 37]. Several typical examples are as follow. (1) Amplitude thresholds: if the unwanted targets are expected to have a lower RCS than the wanted targets, the amplitude of the unwanted returns can be expected to be lower than that of the genuine targets. In this case, an amplitude threshold can be set in the radar causing the echoes below a given amplitude to be ignored. However, wind turbines usually have a RCS larger than some wanted targets, so this method has a very limited utility. (2) Doppler discrimination: it is used to discriminate between wanted moving targets and unwanted static or slow-moving targets that do not exceed a given radial velocity. However, wind turbines are problematic since the blade tip may have a high speed, similar to the speed of a light aircraft or helicopter. (3) Constant false alarm rate (CFAR) [38]: CFAR is designed to maintain radar performance in areas where there are clutter. However, if a desired target within the area has a weaker return than the clutter, or if it stays within the area for several antenna sweeps, the clutter threshold will eliminate the genuine target as well as the clutter. Thus, this technique is not suitable for mitigating the effects of wind farms on radar systems.

Although it may be possible to modify wind farm to reduce their radar signature, it would be much easier to do so if efficient mitigation algorithms can be applied. However, literature search reveals that little work has been published. Even less work effort has been placed on the mitigation techniques without modifications of the wind farm and radar hardware. As such, we present a transponder-based mitigation technique. Note that transponders are widely used for calibrating high-resolution imaging radars. Weiß [39] proposed a novel transponder, which yields a retransmission signal with two additional Doppler frequency shifts. The retransmitted signal can decouple the transponder from the background scatterers, so that it can be used to calibrate synthetic aperture radar (SAR) images at a low cost. In fact, this transponder has extensive applications [40] not just for radiometric calibration. This paper extends it to mitigate the effects of wind farms on radar systems.

## 3. Detecting Wind Turbine Clutter

**Figure 1:**
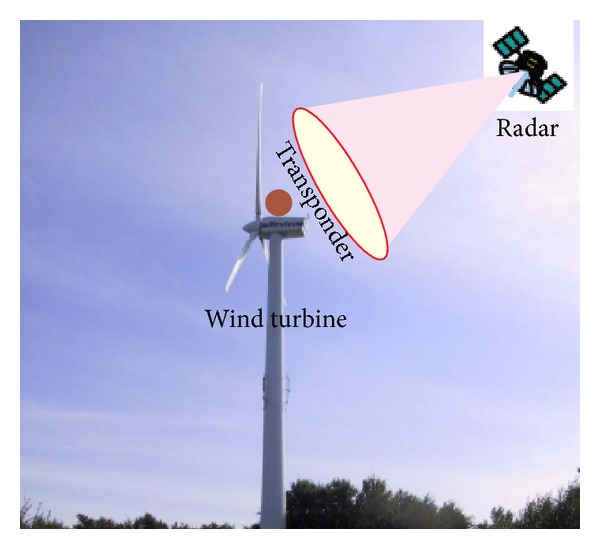
Transponder-based wind turbine clutter mitigation scheme.

**Figure 2:**
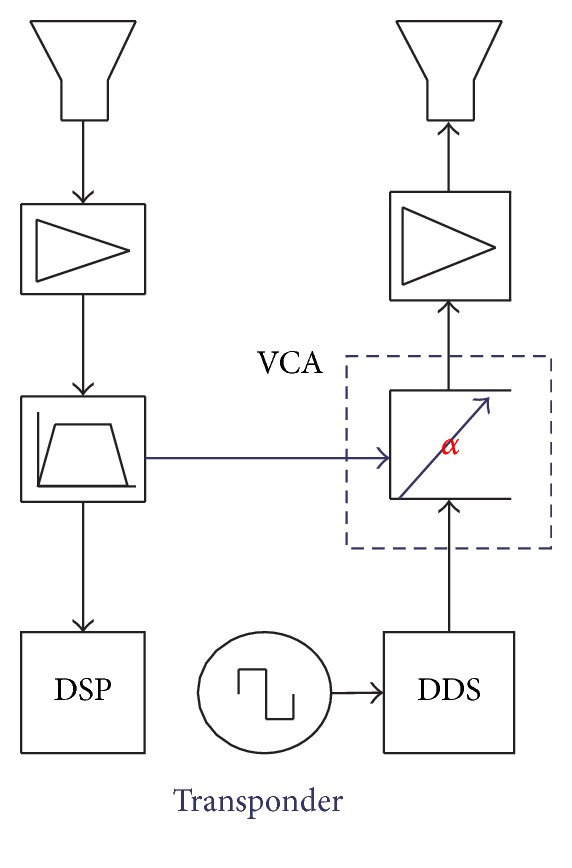
Block diagram of the transponder, DSP: digital signal processor, DDS: direct digital synthesizer.

Figure 1 shows the transponder-based mitigation scheme. The signals received by the transponder are modulated before being retransmitted. As shown in Figure 2, the transponder consists of a low-noise amplifier followed by a controller. The VCA is used to modulate the radar signal in a manner that the retransmitted signal shows two artificial Doppler frequency shifts, one positive shifted and one negative shifted, and the original radar signal. Thereafter, the signal will be amplified to an appropriate level and retransmitted towards the radar receiver. Additionally, to minimise cross-coupling interferences, two omnidirectional antennas are used, one for receive and one for transmit. In this way, if the artificial Doppler frequency shifts are chosen to be larger than the desired Doppler bandwidth of the radar raw data, the transponder signal can be separated during subsequent radar signal processing, allowing for detecting or locating the wind turbines and mitigating its effects on the radar. Note that the transponder is inactive except that it receives some signals being stronger than a given threshold, so that there is no interference to other radar and communication systems.

As the transponder can be seen as an amplitude modulator, the retransmitted radar signal modulated by the transponder can be represented by [39]

(1) $$\begin{array}{c} s_{\mathrm{tr}}\left(t\right)=\left[\alpha +\beta \cos\left(2\pi f_{m}t+\varphi_{m}\right)\right]\cdot s_{o}\left(t\right), \end{array}$$

where *α* and *β* are the attenuation tuning parameters, *f*_*m*_ is the modulation frequency, and *φ*_*m*_ and *s*_*o*_(*t*) denote the starting phase and the transmitted radar signal, respectively. The signal *s*_*o*_(*t*) can be thought as the echo of the transponder without the amplitude modulation. Applying Fourier transform to (1), we have

(2) $$\begin{array}{c} S_{\mathrm{tr}}\left(f\right)=\alpha S_{o}\left(f\right)+\frac{\beta }{2}e^{-j\varphi_{m}}S_{o}\left(f+f_{m}\right)+\frac{\beta }{2}e^{j\varphi_{m}}S_{o}\left(f-f_{m}\right). \end{array}$$

**Figure 3:**
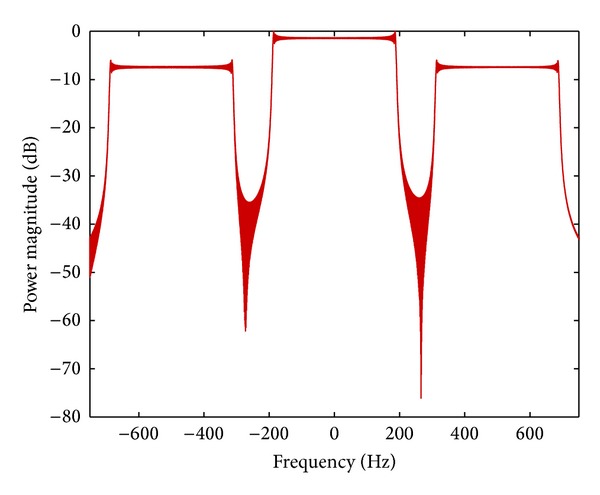
Spectrum retransmitted radar signal modulated by the transponder.

Taking a linear frequency modulated (LFM) signal which is widely used in various radar systems as an example, Figure 3 shows the corresponding retransmission signal in frequency domain. We can see that the retransmitted radar signal shows the original radar signal and two additional frequency shifted signals, one positive and one negative.

The signals coming to the radar receiver can be represented by

(3) $$\begin{array}{c} s_{r}\left(t\right)=s_{u}\left(t\right)+\left[\alpha +\beta \cos\left(2\pi f_{m}t+\varphi_{m}\right)\right]\cdot s_{o}\left(t\right), \end{array}$$

where *s*_*u*_(*t*) denotes the normal radar echoes unmodulated by the transponder. Applying Fourier transform to (3), we have

(4) Sr(f)=Su(f)+αSo(f)+β2e−jφmSo(f+fm)+β2ejφmSo(f−fm).

**Figure 4:**
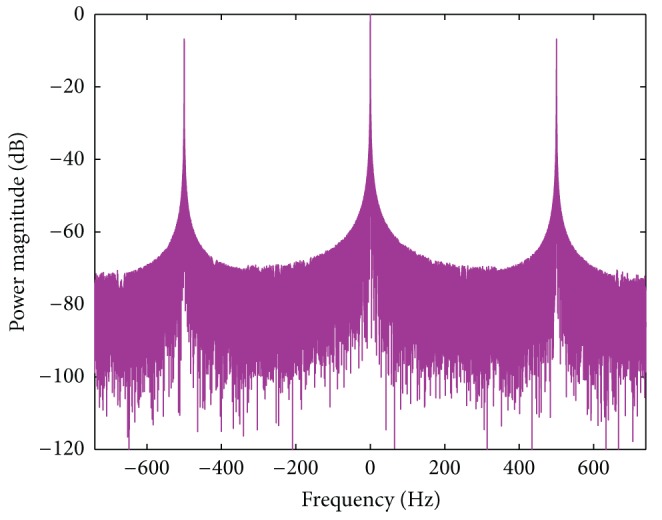
The received transponder signal after range compression.

After range compression with the reference function

(5) $$\begin{array}{c} H_{o}\left(f\right)={S_{o}}^{*}\left(f\right), \end{array}$$

where  * is the conjugate operator, we can get the results like Figure 4.

The upper and lower side bands of this signal can then be acquired using the appropriate filters [39]:

(6a) $$\begin{array}{c} H_{\text{up}}\left(f\right)=\text{rect}\left(\frac{f-f_{m}}{B}\right), \end{array}$$

(6b) $$\begin{array}{c} H_{\text{dn}}\left(f\right)=\text{rect}\left(\frac{f+f_{m}}{B}\right), \end{array}$$

where rect(·) denotes a gate function and *B* is the filter bandwidth which has to be chosen according to the signal bandwidth of the *s*_*o*_(*t*) and the modulation frequency *f*_*m*_. Then, the upper and lower side bands are represented, respectively, by

(7a) $$\begin{array}{c} S_{\text{up}}\left(f\right)=\frac{\beta }{2}e^{j\varphi }S_{o}\left(f-f_{m}\right), \end{array}$$

(7b) $$\begin{array}{c} S_{\text{dn}}\left(f\right)=\frac{\beta }{2}e^{-j\varphi }S_{o}\left(f+f_{m}\right). \end{array}$$

The starting phase *φ*_*m*_ can then be calculated from

(8) $$\begin{array}{c} S_{\text{up}}\left(f+f_{m}\right)\cdot S_{\text{dn}}^{*}\left(f-f_{m}\right)=\beta e^{j2\varphi_{m}}, \end{array}$$

modulo *π*. Using this starting phase, we can get [39]

(9) $$\begin{array}{c} S_{o}\left(f\right)=\frac{\left[S_{\text{up}}\left(f+f_{m}\right)e^{-j\varphi_{m}}+S_{\text{dn}}\left(f-f_{m}\right)e^{j\varphi_{m}}\right]}{\beta }. \end{array}$$

Additionally, the unmodulated signal *S*_un_(*f*) can then be obtained by

(10) $$\begin{array}{c} S_{\text{un}}\left(f\right)=S_{r}\left(f\right)-S_{\text{up}}\left(f\right)-S_{\text{dn}}\left(f\right). \end{array}$$

Thereafter, *s*_*o*_(*t*) and *s*_un_(*t*) can be obtained by inverse Fourier transforms. Note that the signals reflected from the wind turbines are also included in the *s*_un_(*t*). Evaluating *s*_*o*_(*t*) and *s*_un_(*t*) leads to an effective solution to mitigate the effects of the wind farms on radar systems.

## 4. Mitigation Processing Algorithm

As wind farms often occupy only a small area, the radar may be impacted only by the wind farms in a very short time; hence, mitigation processing in the whole radar operation will be redundant and cost inefficient (but most current mitigation techniques just do so). Thus, it is desirable for the mitigation processor to operate only when the radar enters into the wind farms. To reach this aim, we deal with two typical radars in separate ways.

### 4.1. SAR Imaging

**Figure 5:**
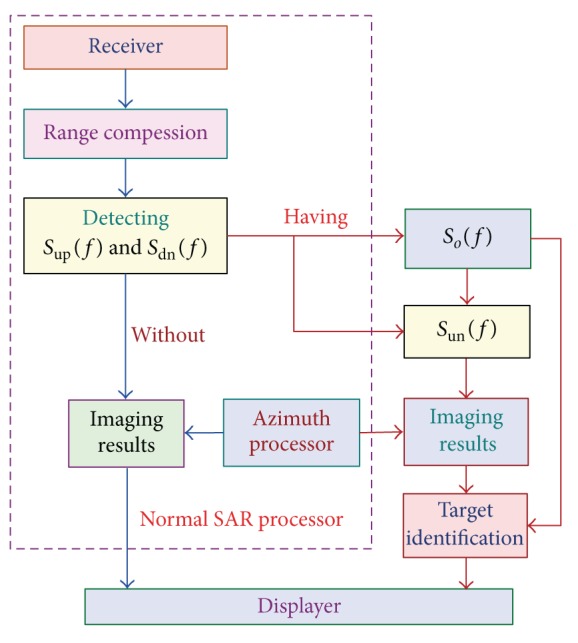
Structure of the mitigation processing for SAR systems.

The first typical example is SAR system, which has been playing a more and more important role in microwave remote sensing.  Figure 5 shows the proposed mitigation processing structure. The received radar signals are range compressed first. We then detect both *S*_up_(*f*) and *S*_dn_(*f*) (see Figure 4). If both *S*_up_(*f*) and *S*_dn_(*f*) are not detected, the subsequent processing steps are the same as the normal SAR azimuth processing steps, without any modification. Otherwise, the mitigation processing method is applied. Firstly, *S*_*o*_(*f*) and *S*_un_(*f*) are extracted using (9) and (10) separately. Next, the imaging results can be obtained by processing *S*_un_(*f*) with the normal azimuth processor. Finally, the “ghost” generated by the wind turbines can be identified and classified by using the transponder as a target of reference.

**Figure 6:**
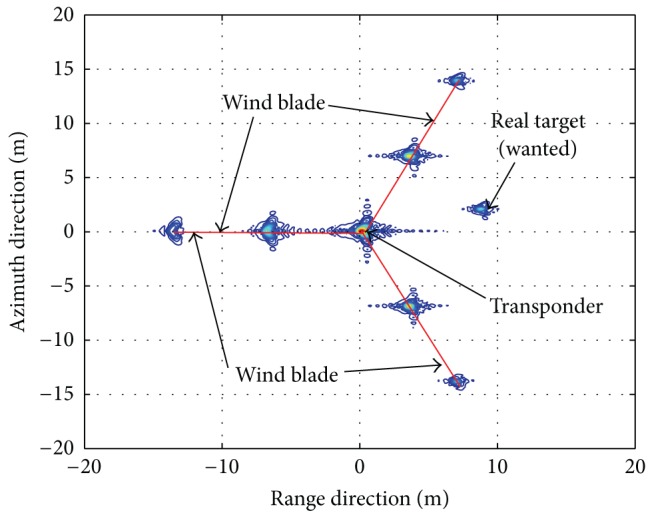
Simulation results for high-resolution SAR system.

As experimental data is unavailable for us, airborne stripmap SAR data is simulated using the parameters listed in Table 1.  Figure 6 shows the processed results with the range-Doppler (RD) imaging algorithm [41]. The transponder can be easily identified because it has high signal-to-noise (SNR) due to the amplitude modulation. As the transponder is placed on the top of the wind mast alike Figure 1, the “ghost” generated by the wind turbines should be symmetrically distributed around the transponder image. Thus, the “ghost” can be identified. Also, in this way, the “true” targets can be identified easily, because the “ghost” should be symmetrically distributed around the transponder image. If the features of the target can be used, the identification and classification performance can be further improved [42]. Once an object has been characterized in this way, this information can be used to select which detections are to be passed to the displayer. The mitigation processor operates only when both *S*_up_(*f*) and *S*_dn_(*f*) have been detected; hence, this mitigation technique is cost efficient, without significantly increasing the burden of the radar processor.

### 4.2. Civil Radar Target Detection

Another typical example is civil radar. Most civil radars will detect any targets which meet the threshold or criteria set in the radar's processor. Its performance is determined by the balance between the probability of detecting wanted targets and the probability of detecting unwanted or false targets. Measures to increase the probability of detecting wanted targets may also increase the probability of detecting false or unwanted targets. Most published work just aims to improve radar's ability to discriminate between wanted and unwanted targets. But, they are not efficient because the mitigation processing is usually applied in the whole radar operation.

Similarly, only when both *S*_up_(*f*) and *S*_dn_(*f*) have been detected, the mitigation processing is necessary. The moving wind turbines, although exhibiting similar characteristics to aircraft, do not of course move their location. Hence, plot filtering (also called plot target extraction) can be used to identify the targets. This is based on analysis of successive returns from a target to determine the direction and speed of its movement. The echoes that do not match the speed characteristics of an aircraft will not be passed to the displayer. The screen displayer has no “raw” radar returns but shows confirmed tracks as a line indicating the direction of the movement and the length of the line indicating the speed of the aircraft.

To further extend plot target extraction so that the track of an aircraft can be projected through a wind farm, here a Kalman filter is applied to obtain an optimal track estimate. A set of parameters describes the target state while the radar passes the wind farm, which can be represented by

(11) $$\begin{array}{c} x\left(t+1\right)=\Phi x\left(t\right)+\Gamma w\left(t\right),\\ y\left(t\right)=Hx\left(t\right)+v\left(t\right), \end{array}$$

where the state vector **x**(*t*) contains the actual target position, velocity, and acceleration at time instance *t*. **y**(*t*) is the observation vector at time instance *t*. **w**(*t*) and *v*(*t*) are the process noise at time instance *t*. Φ and *H* are the system matrixes which describe the deterministic state processes. We assume that both **w**(*t*) and *v*(*t*) are Gaussian distributed noises with expectation 0 and covariance matrix *Q*(*t*) and *R*(*t*), respectively. The subsequent *N*-step smoothing result is

(12) $$\begin{array}{c} \hat{y}\left(t\;|\;t+N\right)=y\left(t\right)-\hat{v}\left(t\;|\;t+N\right),\;N>0, \end{array}$$

with

(13) v^(t ∣ t+N)=v^(t ∣ t+N−1)+Mv(t ∣ t+N)ε(t+N),N=1,2,3,…,

where *M*_*v*_(*t* | *t* + *N*) and *ε*(*t* + *N*) are the Kalman smoothing gain and new information, respectively.

**Figure 7:**
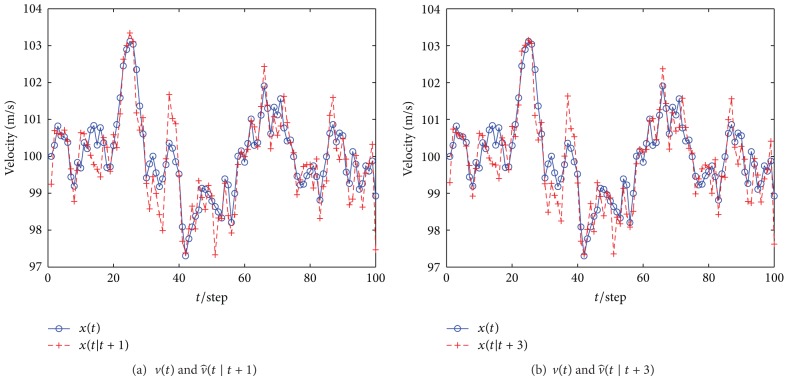
Velocity estimate using Kalman smoothing filter.

**Figure 8:**
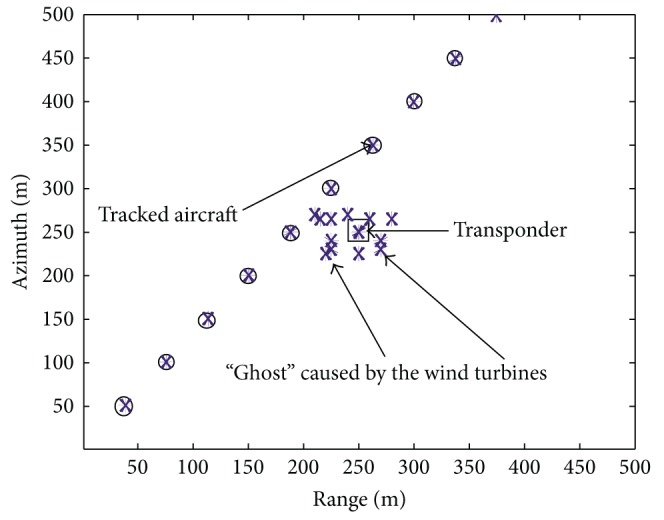
Plot filtering method tracking an aircraft close to a wind farm.

We consider an example ground-based pulse Doppler radar system. The simulation parameters are listed in Table 2. Taking velocity estimation as an example, Figure 7 shows the comparative performances between different steps. As the transponder does not move from its location, once the transponder is identified, we can obtain successful plot filtering results, as shown in Figure 8. In this way, the effects of wind farms on civil radars can be mitigated successfully. This method is particularly effective at differentiating wind turbines from aircraft. In this way, intermittent detection, if associated with stationary objects, can be removed from the information passed to the displayer.

## 5. Conclusion

It is well recognized that wind farms may cause degradation to many radar applications. Mitigation processing is thus necessary, but little work has been published. As such, this paper proposes a transponder-based mitigation technique. Note that the transponder is not a new concept, which has been proposed for calibrating high-resolution imaging radars. This paper uses the transponder to determine whether the radar is impacted by the wind farms. If so, the effects of wind farms on radar systems can then be mitigated by the presented processing algorithms. Note that Kalman smoothing filtering and plot target extraction are used in this paper; practically, other advanced filtering techniques or target detection algorithms may be also feasible. Because experimental data is not available for us, simulation data is used to validate this transponder and signal processing algorithm combined mitigation technique. An advantage is that, as the mitigation processor can operate only when the radar enters into the wind farms, this technique can be implemented at a low cost, without significantly increasing the burden of the radar processor. The originality of this paper lies in the transponder and mitigation algorithm combined wind farm mitigation technique. Future wind farms may be constructed with new materials that are less reflective in radar frequency bands. But this trend will not change the nature of the problems that turbines potentially cause to radars. In a world where renewable energy sources will become increasingly necessary, the transponder-based mitigation technique can provide a potential solution.

## Figures and Tables

**Table 1:** Simulation parameters.

Parameters	Values	Units
Carrier frequency	1.25	GHz
Pulse repetition frequency	1000	Hz
SAR flying velocity	180	m/s
SAR flying altitude	6000	m
Chirp period	1 × 10^−6^	s
Azimuth resolution	1	m
Range resolution	1	m

**Table 2:** Simulation parameters.

Parameters	Values	Units
Carrier frequency	10	GHz
Pulse repetition frequency	12000	Hz
Pulse duration	2 × 10^−6^	s
Pulse width	50	MHz
Antenna beam width	3°	s
Doppler processor	Fast Fourier transform (FFT)	m
